# Transient systolic anterior motion in a patient with junctional rhythm in the intensive care unit

**DOI:** 10.2478/jccm-2025-0021

**Published:** 2025-07-31

**Authors:** Alfred Ibrahimi, Saimir Kuci, Ormir Shurdha, Romina Teliti

**Affiliations:** Division of Intensive Care, Mother Teresa Hospital, Tirana, Albania; Division of Cardiology, Mother Teresa Hospital, Tirana, Albania; Division of Cardiology, German Hospital International, Tirana, Albania

**Keywords:** systolic anterior motion, mitral valve regurgitation, junctional rhythm, transesophageal echocardiography, right ventricular pacing

## Abstract

Systolic anterior motion (SAM) of the mitral valve refers to the unusual movement of the anterior and sometimes the posterior mitral valve leaflets toward the left ventricular outflow tract (LVOT) during systole. This phenomenon is most frequently associated with the asymmetric septal variant of hypertrophic cardiomyopathy (HCM), but it can also occur in conditions like acute myocardial infarction, diabetes mellitus, hypertensive heart disease, after mitral valve repair, and even in asymptomatic individuals during dobutamine stress tests. We present a case of transient SAM induced by a junctional rhythm along with high doses of dobutamine and nitroglycerin in an intensive care unit (ICU) setting. Transesophageal echocardiography (TEE) played a crucial role in detecting SAM and showed that transitioning from a junctional rhythm to a ventricular paced rhythm led to an improvement in the SAM condition.

## Introduction

Systolic anterior motion (SAM) of the mitral valve refers to the unusual movement of the anterior and, less frequently, the posterior leaflets of the mitral valve toward the left ventricular outflow tract (LVOT) during systole. This motion is primarily associated with the asymmetric septal variant of hypertrophic cardiomyopathy (HCM), but it has also been observed in conditions such as acute myocardial infarction, diabetes mellitus, hypertensive heart disease, after mitral valve repair, and even in asymptomatic individuals during dobutamine stress tests.

## Case presentation

A 65-year-old man, was presented in regional hospital emergency due to an episode of syncope. Physical examination was notable for severe bradycardia, elevated values of blood pressure (BP) (230/100 mmHg) and persistent low oxygen saturation on pulse oximetry (89%). The initial electrocardiogram showed a heart rate of 35 bpm, junctional rhythm. He referred dizziness and weakness in the last two months. His past medical history was notable for hypertension, diabetes and obesity. The patient was firstly managed in emergency of the regional hospital. To increase the heart rate, atropine was administered alongside dopamine and dobutamine infusions. High doses of i.v. nitroglycerin were also used, adjusted according to BP levels. After starting the drugs, the heart rate was increased 50–60 bpm (junctional rhythm), the BP was stabilized, but clinical situation was worsened. The patient entered in an oligo-anuric state, so i.v. furosemide was injected in order to increase the urine output.

The progressive deterioration of the patient's condition and the lack of a catheterization laboratory were the reasons why the patient was transferred in our institution for further treatment.

**Fig. 1.**
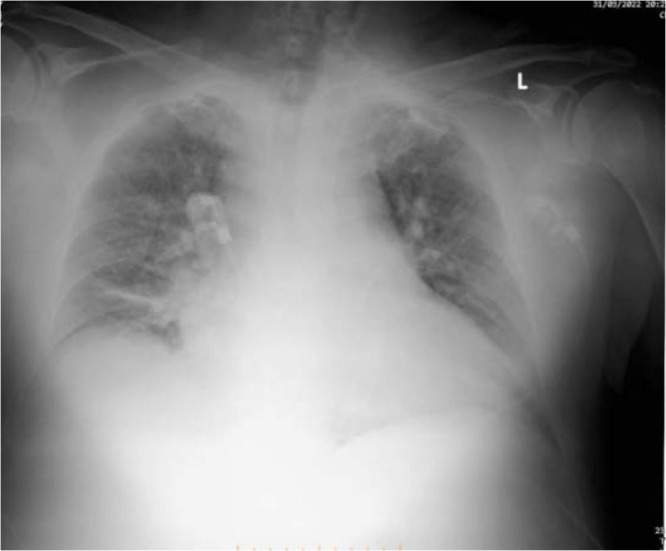
Thoracic X Ray, bilateral opacification

He had derangement of his lab workup, as follows: Creatinine (Cr) of 2.23 (0.55 to 1.30 mg/dL normal), blood urea nitrogen (BUN) of 100 (14 to 39 mg/dL normal), Glycemia 303 mg/dL, Troponin I 0.218 (< 1.0 ng/mL normal). His blood count (CBC) showed WBC 10.42x103qe/μl, RBC 3.75x105qel/μl, Hb 10.7 g/dL. An arterial blood gas (ABG) a pH of 7.31, hyperkalemia K+of 6.54 mEq/L (3.8–5.0 mEq/L normal), base excess (BE) of −11.1, (−2/+2 normal). The thoracic X-Ray revealed opacification of both lungs, probably pulmonary edema. *Figure 1*

### Treatment

**Fig. 2.**
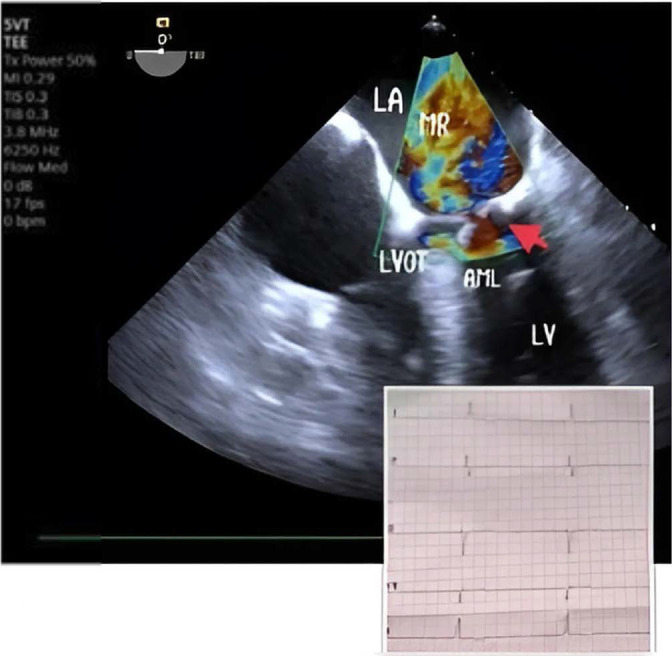
Severe mitral regurgitation in junctional rhythm. MR mitral regurgitation, LA left atrium, LV left ventricle, LVOT left ventricular outflow tract, AML anterior mitral leaflet

Upon admission to our hospital, the patient was experiencing severe respiratory failure and was promptly transferred to the intensive care unit (ICU). Oxygen saturation levels fell to 50% despite ventilation support through a face mask. Given this critical condition, the decision was made to intubate the patient for mechanical ventilatory support. Unfortunately, even with this support, oxygen saturation did not improve, although blood pressure stabilized at an acceptable level of 100/60 mmHg. Due to a poor transthoracic echocardiogram (TTE) window, a transesophageal echocardiogram (TEE) was performed, which SAM of the mitral valve with severe mitral regurgitation (MR). *Figure 2* Pressure gradient across the LVOT was 50 mmHg and the basal interventricular septum thickness was 12,5 mm. No segmental or global wall motion abnormalities were observed.

**Fig. 3.**
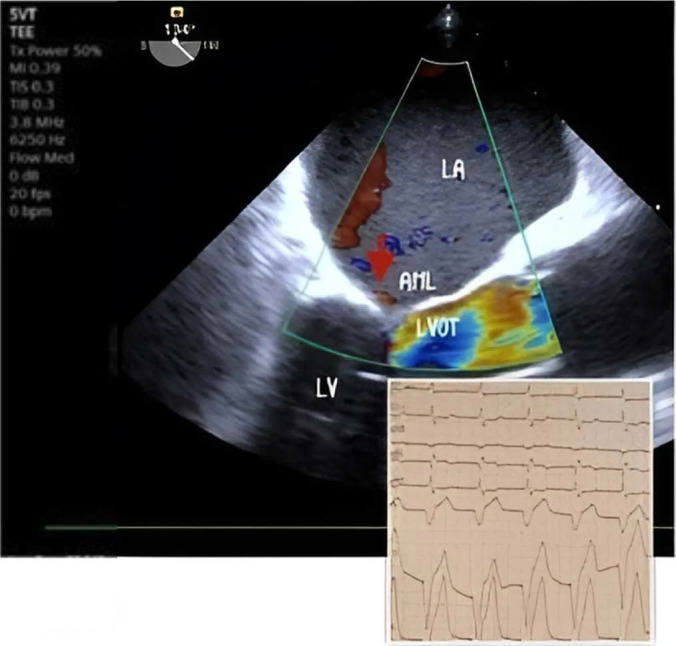
Reverse of mitral regurgitation after temporary Pacemaker implantation. LA left atrium, LV left ventricle, LVOT left ventricular outflow tract, AML anterior mitral leaflet

Following this, intravenous nitroglycerin was discontinued, and intravenous fluids along with noradrenaline were administered, but there was no notable improvement in saturation over the next 20 minutes. Correction of bradycardia and optimization of preload and afterload failed to resolve SAM and MR. Consequently, a temporary right ventricular (RV) transvenous pacing was implanted via the right subclavian vein (ventricular pacing frequency 90 bpm) in the ICU. This intervention led to a rapid improvement; MR severity decreased from severe to mild, no LVOT obstruction and good urine output was noted within five minutes. *Figure 3*

Within the first few hours, mixed acidosis (both respiratory and metabolic) and hyperkalemia were corrected. The patient was extubated after 12 hours, displaying stable respiratory and cardiac parameters.

**Fig. 4.**
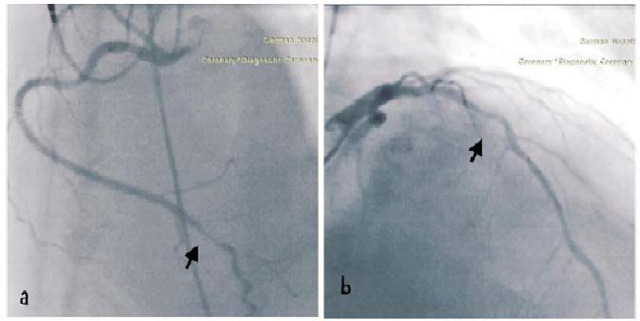
(a) Right coronary artery (RCA) distal tract stenosis (90%), (b) Left Anterior descendent artery (LAD) medial tract stenosis (75–90%)

The following day, a permanent dual-chamber pacemaker (DDDR) was implanted. After 48 hours of intravenous fluids to mitigate contrast-induced nephropathy risk, a coronary angiogram was performed. This revealed 90% stenosis in the distal tract of the right coronary artery (RCA) and 75–90% stenosis in the proximal tract of the left anterior descending artery (LAD). Both stenoses were addressed with percutaneous coronary intervention using two drug-eluting stents. *Figure 4*

The patient was discharged after six days of hospitalization in good condition.

## Discussion

This case report emphasizes the occurrence of SAM triggered by junctional rhythm, along with the use of high doses of dobutamine and nitroglycerin in the ICU. It also underscores the significance of TEE for rapid diagnosis of SAM, especially when TTE is inadequate.

Originally, SAM was associated primarily with HCM. However, literature has shown that SAM can occur in various contexts beyond HCM [1,2], such as after mitral valve repair [3,4], in patients with diabetes mellitus, and during catecholamine stimulation [5,6,7], particularly with dobutamine [8]. Other contributing factors include volume depletion [8] and junctional rhythm [3,4]. Conditions that alter left ventricular (LV) geometry or septal wall motion, such as bulging of the subaortic septum can also lead to SAM [9,10]. In non-HCM cases, SAM is more likely under certain physiological conditions, such as reduced preload, decreased afterload and increased inotropic state [10].

Patients with diabetes may develop SAM due to left ventricular hypertrophy, resulting from heightened sensitivity to β-adrenoreceptors [11]. Dobutamine stress echocardiography has been shown to induce SAM by changing the anatomical characteristics of the mitral valve [12]. Alterations in LV geometry from opposing hyperkinetic and hypokinetic regions after a myocardial infarction, can also lead to SAM, which is clinically relevant because vasodilators and inotropic agents used in cardiogenic shock may exacerbate hemodynamic instability [9]. Additionally, general anesthesia can trigger SAM due to its vasodilatory effects, even in the absence of cardiac abnormalities [10]. SAM may cause unexpected cardiopulmonary complications during surgery, particularly when myocardial hypertrophy or states of hypovolemia and anesthesia-induced vasodilation are present, potentially leading to misdiagnosis as cardiogenic pulmonary edema [13]. Junctional rhythm worsens SAM, MR, and LVOT obstruction by causing atrioventricular dyssynchrony and loss of atrial kick, leading to decreased cardiac output and mean arterial pressure [3].

In our case, the patient presented with junctional bradycardia at a heart rate of 30 bpm, which increased to 50–60 bpm following continued dobutamine administration. Clinical signs—including dyspnea, desaturation, orthopnea, and crackles on auscultation—suggestive of fluid overload, led to in inappropriate treatment with vasodilators and diuretics. The patient's progressive respiratory distress necessitated intubation and TEE, which revealed hypovolemia, a hyperdynamic LV, SAM, and severe MR.

A few cases in the literature report similar instances of SAM induced by junctional rhythm and dobutamine. Management strategies for SAM depend on the underlying causes and severity. Generally, reducing or discontinuing inotropic agents and administering vasopressors can effectively resolve SAM [3]. In our patient, halting dobutamine and continuing vasopressors and volume replacement did not improve SAM or MR. It was only after the implantation of a temporary transvenous right ventricular pacemaker that the severity of MR decreased from severe to mild, making more vulnerable that junctional rhythm was likely the main predisposing factor that deteriorated SAM. However, it cannot be said with certainty that junctional rhythm precipitated this patient in SAM. Also, it is not clear the precise mechanism by which RV pacing reduced MR associated with SAM. It is hypothesized that a delayed contraction of the inter-ventricular septum could lead to dyssynchrony between the right and left ventricles, possibly reducing the suction effect that draws the mitral valve anteriorly. While there are several studies about dual or RV pacing in HCM, research specifically examining the use of pacing for managing non-HCM related SAM remains limited. In a previous article, SAM after mitral valve plasty was resolved with LV pacing, while atrial and RV pacing were unsuccessful, speculating that the reduction of left ventricular dyssynchrony resulted in resolution of MR associated with SAM [4]. Further investigations with a larger number of subjects could clarify the effects of ventricular pacing in non-HCM.

## Conclusion

This case highlights junctional rhythm as a major pre-disposing factor for SAM in the ICU, compounded by the effects of high doses of dobutamine and nitroglycerin. It underscores the challenges of treatment and emphasizes the need for future research on the role of pacing and hemodynamic optimization in SAM management. Furthermore, it highlights the important role of TEE in the rapid diagnosing of SAM when TTE imaging is suboptimal.
